# End-to-End Network Intrusion Detection Based on Contrastive Learning

**DOI:** 10.3390/s24072122

**Published:** 2024-03-26

**Authors:** Longlong Li, Yuliang Lu, Guozheng Yang, Xuehu Yan

**Affiliations:** 1 College of Electronic Engineering, National University of Defense Technology, Hefei 230037, China; lilongs@nudt.edu.cn (L.L.);; 2 Anhui Province Key Laboratory of Cyberspace Security Situation Awareness and Evaluation, Hefei 230037, China

**Keywords:** network intrusion detection, contrastive learning, CNN, GRU, end-to-end

## Abstract

The network intrusion detection system (NIDS) plays a crucial role as a security measure in addressing the increasing number of network threats. The majority of current research relies on feature-ready datasets that heavily depend on feature engineering. Conversely, the increasing complexity of network traffic and the ongoing evolution of attack techniques lead to a diminishing distinction between benign and malicious network behaviors. In this paper, we propose a novel end-to-end intrusion detection framework based on a contrastive learning approach. We design a hierarchical Convolutional Neural Network (CNN) and Gated Recurrent Unit (GRU) model to facilitate the automated extraction of spatiotemporal features from raw traffic data. The integration of contrastive learning amplifies the distinction between benign and malicious network traffic in the representation space. The proposed method exhibits enhanced detection capabilities for unknown attacks in comparison to the approaches trained using the cross-entropy loss function. Experiments are carried out on the public datasets CIC-IDS2017 and CSE-CIC-IDS2018, demonstrating that our method can attain a detection accuracy of 99.9% for known attacks, thus achieving state-of-the-art performance. For unknown attacks, a weighted recall rate of 95% can be achieved.

## 1. Introduction

The past two decades have witnessed the rapid development of network technology. Especially in recent years, the application of 5G technology has landed, making it possible to connect everything. In 2020, the number of active Internet of Things (IoT) devices worldwide reached 15.1 billion, and this number is expected to grow to 29.42 billion by 2030 (https://www.statista.com/statistics/1101442/iot-number-of-connected-devices-worldwide/, accessed on 21 March 2024). Accordingly, cyber threats are becoming more serious. The network intrusion detection system (NIDS) is one of the most important types of security devices in cyberspace and has been widely used in safeguarding various information systems. A NIDS continuously monitors incoming and outgoing network traffic, analyzing it to detect cyberattacks [1].

In general, network intrusion detection methods can be broadly classified into two main categories: signature-based IDS and anomaly-based IDS [2,3,4,5]. Signature-based IDS involves the generation of signatures for known attacks, which are then compared against incoming data instances [6,7,8]. These methods offer the advantages of high detection precision and a low false alarm rate; however, they may be limited in their ability to detect novel attacks. On the other hand, anomaly-based IDS focuses on detecting deviations from expected behaviors by constructing models to assess benign behavior and issuing warnings when a given instance deviates significantly from typical behavior beyond a predefined threshold.

Anomaly-based methods have garnered increased attention due to their superior capabilities in detecting unknown attacks. A significant trend in anomaly-based IDS is the utilization of machine learning for its robust representation capabilities. As network traffic becomes increasingly complex, a primary challenge lies in designing a general and stable representation model that can effectively distinguish anomalies from benign traffic.

Firstly, relying on feature-ready datasets limits the generalizability and feasibility of machine learning-based approaches, which often require feature engineering for typical attacks. Traditional machine learning models, such as support vector machines, decision trees, and K-nearest neighbors, are trained to acquire detection capabilities using these features [9,10,11]. Deep learning has also been applied to construct IDSs in recent years. However, many methods still depend on feature-ready datasets [5,12,13,14]. The advantages of building models for feature-ready data are semantically interpretable and easy to train. However, the cost of feature engineering also significantly increases with the number of attack types. The designed features also lose some information. Therefore, some methods attempt to construct automatic feature extraction models [15,16] for the raw traffic. We prefer automatically extracting features from raw traffic because such methods can fully leverage the powerful feature extraction capabilities of deep learning and are more feasible for new types of attacks.

Secondly, the boundary between benign and malicious traffic is becoming increasingly difficult to determine. Attackers are continuously upgrading malware to alter their communication behaviors in order to bypass detection. New attack variants will likely be challenging to detect using previous features, necessitating new feature engineering. Deep learning-based approaches also encounter the issue of adversarial samples. Attackers can treat NIDS as a black-box system and continuously query and adjust the attack traffic features based on feedback until they successfully bypass the NIDS [17,18]. In order to enhance the capability of IDS to differentiate between benign and malicious traffic, it is crucial to develop models that accentuate the distinction between benign and malicious samples.

In this paper, we propose CHCG, a contrastive learning-based end-to-end intrusion detection framework with hierarchical Convolutional Neural Networks (CNN) and Gate Recurrent Unit (GRU) networks to extract features from raw traffic. CHCG has the capability to directly process network traffic PCAP data, eliminating the need for supplementary feature engineering. The raw bytes in the packet sequence are used as the input. The bytes of a single packet are converted into a two-dimensional matrix, from which the spatial feature vector is then extracted using CNN. The spatial feature vectors of all packets in the sequence are composed into a vector sequence. The temporal features are subsequently extracted using GRU to produce a representation that encompasses both spatial and temporal features. Contrastive learning encourages the model to encode benign traffic into a compact cluster within the representation space and amplifies the distinction between benign and malicious representations. This, in turn, helps widen the margin of the decision boundary. Finally, the cosine similarity between the representations of the testing and benign traffic is used as the basis for detection. Experiments show that the proposed method exhibits state-of-the-art performance and high accuracy against unknown attacks. Our main contributions are summarized as follows:

1. We design a hierarchical model that combines CNN and GRU networks for the autonomous extraction of spatiotemporal features from raw traffic data. Furthermore, an embedding layer is integrated to enhance the representation dimension of the protocol header bytes, which contain more important information.
2. We propose an intrusion detection framework based on contrastive learning. Contrastive loss can offer a wider decision boundary margin compared to cross-entropy loss. Using cosine similarity with benign traffic representations as the basis of detection can lead to higher accuracy and a lower false alarm rate. Moreover, unknown attacks can be detected.
3. We introduce the granularity of IP pairs for packet collection. Compared with flow-level, IP pair granularity can present inter-flow characteristics, which has advantages for discovering attack traffic that looks normal in a single flow, such as Dos and PortScan.
4. The proposed framework undergoes evaluation using widely utilized datasets, namely, CIC-IDS2017 [19] and CSE-CIC-IDS2018 [20]. The results demonstrate that our approach can attain a detection accuracy of 99.9% for known attacks, thus achieving state-of-the-art performance. For unknown attacks, a weighted recall rate of 95% can be achieved.

The rest of paper is organized as follows: Section 2 introduces related works. Section 3 describes the methodology used in our research. Section 4 demonstrates the experiments using CIC-IDS2017 and CSE-CIC-IDS2018 datasets. Section 5 concludes this paper.

## 2. Related Work

This section presents the related works on intrusion detection in recent years and mainly focuses on deep learning methods.

Some studies have concentrated on the development of novel features or algorithms for feature selection. Mirsky et al. proposed Kitsune, an online network IDS [21]. Kitsune utilizes a set of autoencoder neural networks with unsupervised learning to differentiate between benign and malicious network traffic. When packets are captured, Kitsune extracts their statistical features and reconstructs them with several auto-encoders. The reconstruction error distinguishes between normal and malicious traffic patterns. Similarly, Li et al. proposed an autoencoder-based IDS and applied feature selection and feature grouping algorithms [12]. The reconstruction error of autoencoders was used to detect anomalies. However, they only considered the CSE-CIC-IDS2018 dataset, so the evaluation was insufficient. Xu et al. proposed an IDS with GRU networks, multilayer perception (MLP), and softmax module, achieving an overall detection rate 99.42% on the KDD 99 dataset and 99.31% on the NSL-KDD dataset [22]. Alzubi et al. combined CNN and LSTM to identify hidden patterns of data, achieving improved accuracy on the CSE-CIC-IDS2018 dataset [14]. Wang et al. designed a feature selection approach and generated 143 features for encrypted traffic. They also proposed a detection framework with long short-term memory (LSTM), ResNet, and XGBoost [23]. The quality of features dramatically influences the performance of the aforementioned methods, indicating that new attacks may be difficult to detect.

End-to-end methods are becoming a hotspot for fully leveraging deep neural networks, which directly learn features from raw traffic data. Wang et al. proposed an encrypted classification method using one-dimensional CNN [24]. The raw bytes of a session or flow are input into the network for automatic feature extraction. They validated the method using the ISCX VPN-nonVPN dataset and demonstrated its effectiveness. Their other work combines CNN and LSTM to learn the packet-level spatial features and the flow-level temporal features [15]. The hierarchical spatiotemporal features have better performance in characterizing network traffic behaviors. This network structure is also utilized in other studies. Lin et al. proposed TSCRNN, an encrypted traffic classification scheme that extracts abstract spatial features using CNN and temporal features using stacked bidirectional LSTM [25]. Differently, they divided all raw bytes by 255 for normalization, which is more efficient than the one-hot encoding in Wang et al.’s work. Shapira et al. proposed FlowPic [26], transforming packet sizes into grayscale images and utilizing CNN for classification. FlowPic has an advantage in processing speed, but the amount of information extracted from the packet is insufficient for handling complex traffic. PBCNN [16] also adopts a hierarchical structure that utilizes CNN to extract abstract features of individual packets and packets within a flow. Generally, a hierarchical feature extraction structure is considered an appropriate choice for representing raw traffic data.

In recent years, contrastive learning has led to significant advances in self-supervised representational learning [27,28,29]. The basic idea is to pull the anchor closer to positive samples in the embedding space and push it away from negative samples. The positive samples are typically data augmentations of the anchor, while the negative samples are randomly selected samples from the minibatch, excluding the anchor and its augmentations. The lack of labels may cause samples of the same class in the minibatch to be incorrectly regarded as negative pairs. Consequently, supervised contrastive learning [30] redefines positive samples as those from the same class and performs better than cross-entropy loss-based methods. In this paper, we apply supervised contrastive learning to develop a representation model for network traffic using labels. Some works have also proposed IDSs based on contrastive learning. FeCo [31] is a federated-contrastive-learning framework that aims to address users’ privacy concerns and develop an accurate model for the benign class. Lopez-Martin et al. [32] represented features and labels within the same vector space. The study aimed to minimize the distance between each label prototype and its sample-class samples. However, the two methods are not end-to-end, and the tested datasets are feature-ready. Yue et al. [33] proposed a heuristic method to generate traffic augmentations and combined contrastive loss and cross-entropy loss to enhance the model training. However, they only tested the performance on raw traffic using a private dataset and still relied on statistical features from public datasets.

Table 1 provides a summary of the related works discussed in the paper. The proposed method can be executed in an end-to-end manner, eliminating the need for feature engineering. An additional benefit is the ability to detect unknown attacks.

## 3. Methodology

This paper proposes a network intrusion detection framework based on contrastive learning and a hierarchical representation model. The framework aims to solve the following problems:

- **How can we automate the extraction of general effective features from raw traffic data without the need for complex feature engineering?**
  Extracting useful features from raw traffic is challenging because network behavior typically involves numerous packets, some of which may be encrypted. Considering the intra- and inter-features of packets, we design a hierarchical structure. We utilize CNN to learn the spatial features of a single packet and GRU networks to learn the temporal features of sequential packets.
- **How can a network intrusion detection model be trained to ensure a significant margin between the decision boundary and the training points?**
  It has been shown that cross-entropy loss results in a much smaller margin between the decision boundary and the training points than optimal. As a result, the trained IDS is sensitive to noisy labels, susceptible to adversarial sample attacks, and challenging to detect unknown attack traffic. In this paper, we employ contrastive loss to represent benign traffic more compactly in the embedding space and separate attack traffic from benign traffic, thereby widening the margin of the decision boundary of the trained model. As a result, the trained model becomes more robust in the presence of noisy labels and adversarial samples, enabling the detection of unknown attacks.

### 3.1. Framework

**Figure 1:**
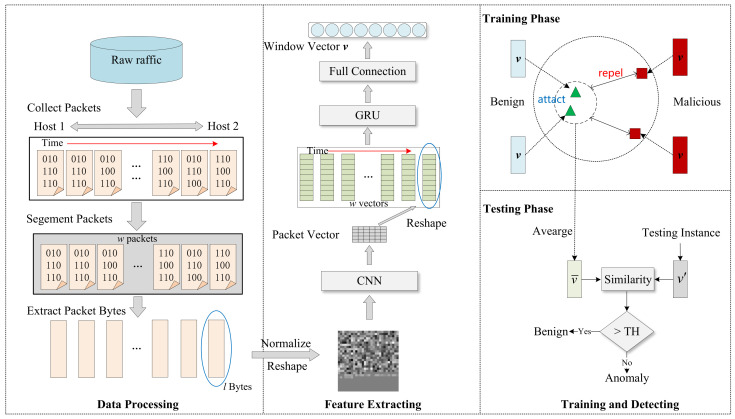
The framework of CHCG.

Most machine learning-based IDS methods detect network anomalies by utilizing features extracted from flows, even though some flows may persist for a considerable duration. Therefore, these methods are more suitable for offline detection. In order to detect malicious traffic at an early stage, we aim to identify a specific number of packets as soon as they are captured. CHCG automates the extraction of raw traffic data features using CNN and GRU models without the need to wait for the flow to finish. This strategy avoids the cost of waiting for the end of a TCP connection and extracting statistical features. The framework of CHCG is shown in Figure 1, including data processing, feature extraction, training, and detection.

- **Data processing.** Upon the arrival of network traffic packets, it is necessary to transform them into a compatible format that can be readily utilized by deep learning models. The packets are grouped either at the flow level or IP pair level and then organized in a sequential order according to their respective arrival times. Subsequently, the raw bytes of the packets are extracted for use as input.
- **Feature extraction.** Taking into account the spatial features of an individual packet and the temporal relationships between packets, we design a hierarchical model based on CNN and GRU for automated feature extraction. In the case of protocol header bytes, an embedding layer is incorporated to enhance the dimension of data representation, rather than solely relying on numerical values.
- **Training.** Contrastive loss is used to promote the formation of a compact cluster for the representation vectors of benign traffic, while simultaneously ensuring a significant separation from the representation vectors of malicious traffic. As a result, the distinction between benign and malicious traffic is magnified in the representation space, leading to increased robustness.
- **Detection.** The core of the benign clusters is determined by calculating the average of the representations of benign flows in the training set. The anomaly score is determined by calculating the cosine similarity between the test representation and the core vector. Consequently, unknown attacks can be identified based on their low similarity to benign traffic.

### 3.2. Data Processing

#### 3.2.1. Capture Packets

Data formats and feature distributions vary significantly across different granularities of network flows. Dainotti et al. [35] summarized five common granularities of network flows, including TCP connections, flows, bidirectional flows, services, and hosts. A flow is typically characterized by the same 5-tuple (source IP, source port, destination IP, destination port, and transport layer protocol) over a period of time. A bidirectional flow is one in which the source and destination of the 5-tuple are interchangeable, and it is also known as a session. Flows and sessions are the most common granularities for network traffic analysis and are typically selected in most studies. The method proposed in this paper uses sessions as one of the detection granularities.

Another granularity used in this paper is IP pairs, also known as host pairs. Dainotti et al. [35] classified host granularity as the predominant traffic generated by a host, under the assumption that bidirectional traffic to and from the host can be observed. We refine this granularity by using the IP address pairs of both communicating parties as the analysis granularity, where source and destination IPs can be exchanged. Thus, it is more relevant than using one host’s IP as the object of detection. Another advantage is that the granularity of IP pairs contains multiple sessions, which can reflect inter-flow characteristics. Especially for PortScan and DoS attacks, their intra-flow characteristics are closer to normal traffic, but the inter-flow characteristics exhibit clear anomalies.

**Figure 2:**
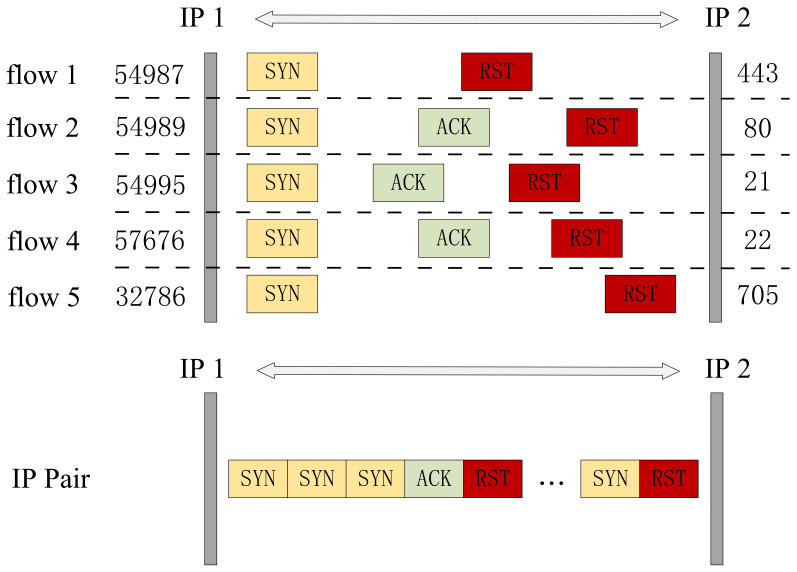
The packets generated by the PortScan attack are displayed at the granularity of flow and IP pair, respectively. Each flow starts with a TCP SYN packet and then receives an ACK or RST, which can also occur in benign flows. However, at the IP pair granularity, a large number of consecutive SYN packets appear within a short period of time, indicating a clear anomaly.

Figure 2 illustrates the benefits of IP pair granularity in detecting PortScan attacks. Looking at just a single flow, such characteristics may also appear in benign traffic. When analyzing multiple flows together, anomalies can be detected immediately. Therefore, IP pair granularity can serve as a complement to flow and session granularity. It offers advantages in detecting attacks with distinct inter-flow characteristics.

For both granularities, the captured packets are organized sequentially, and the time interval between packets should not be too long. If no new data packets are received within a certain period, such as 2 min, the packet collection process of a session or an IP pair should be halted, and the cache should be cleared to avoid unnecessary occupation of storage. It is reasonable to infer that prolonged waiting signifies the termination of previous communication. Assuming there are *n* collected packets from the same 5-tuple or IP pair, where $p_{i}$ represents the *i*-th packet, the packet sequence is represented as $P=[p_{1},p_{2},\ldots ,p_{i},\ldots ,p_{n}]$.

#### 3.2.2. Segment Packets According to a Fixed Window Size

Now, the collected packets are segmented based on a fixed window size, denoted as *w*. The objective of segmentation is twofold: to timely identify incoming traffic for alleviating storage constraints and to align the input for the deep learning model. For the session granularity, we select the first *w* packets within a session.

For the IP pair granularity, the detection window slide over the packet sequence. The sequence within a window can be denoted as $P^{\left(w\right)}=\left[p_{i},p_{i+1},\ldots ,p_{i+w}\right]$. When the sequence has been processed, the window slides to the next position. We define *s* as the slide step and typically set its value to half of the window size. Before generating a new sequence, we check whether the number of remaining packets is more than *s*. If not, it is believed that the remaining data packets are too small to warrant further detection. If the number is greater than *s*, a new window sequence is generated, and empty packets are used to make up the shortfall when the remaining packets are insufficient to form a window. Then we obtain the new sequence $P^{{\left(w\right)}^{\prime }}$.

(1) $$P^{{\left(w\right)}^{\prime }}=[p_{i+s},p_{i+s+1},\ldots ,p_{i+s+w}]$$

#### 3.2.3. Extract Packet Data

Raw traffic data usually refers to all the bytes contained in a packet. For PCAP-formatted traffic, the bytes in each packet can be easily separated. These bytes contain the protocol headers and payloads for each layer of the network protocol and cover all communication information. Many studies have selected raw packet data as inputs and demonstrated satisfactory performance [15,25,36]. The advantages include lower costs for feature engineering and faster data processing speed. To minimize distractions, we remove the bytes associated with the MAC address and IP address. It is evident that such private information typically varies with each attack; otherwise, an address filter can effectively block the attack. Moreover, most artificial datasets have a predetermined attack trace or are anonymized due to privacy concerns.

**Figure 3:**
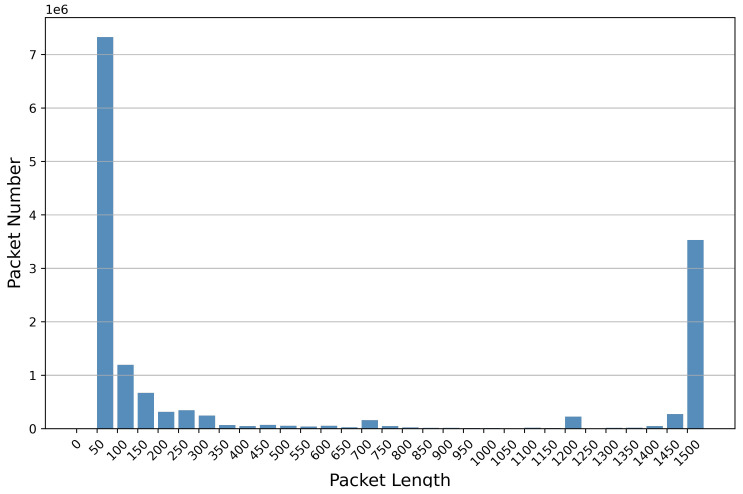
CSE-CIC-IDS2018 packet length distribution (2% random sampling). The x-axis coordinates represent intervals with packet lengths ranging up to 50. Each packet number bar contains the minimum value of the interval, not the maximum value.

Therefore, our approach takes the first *l* bytes from the packet, excluding the MAC and IP addresses, as the input for feature learning. If the packet has insufficient bytes, it will be padded with 0 × 00. Finally, we obtain the processed result, denoted as $P_{l}^{\left(w\right)}$. We sampled approximately 2% of the PCAPs from CSE-CIC-IDS2018 and analyzed the distribution of packet lengths, as illustrated in Figure 3. All the packets are within 1500 bytes, which is the Maximum Transmission Unit (MTU) of Ethernet. The percentage of 1500 bytes is 23.75%. Therefore, we set *l* as 1480 after removing IP and MAC addresses.

### 3.3. Feature Extraction

In this part, we aim to learn the representation of spatiotemporal features of the packet sequence. We first utilize CNN to learn the spatial features of each packet and obtain a feature vector. The output vectors of packets in the detection window form a sequence of vectors, serving as the input for GRU networks to learn temporal features. The output of the representation model is an m-dimensional vector, denoted as v.

#### 3.3.1. Inputs

After data processing, we obtain $P_{l}^{\left(w\right)}$, the object of feature extraction. It can be formalized as:

(2) $$P_{l}^{\left(w\right)}=\left[a_{1},a_{2},\cdots ,a_{i},\cdots ,a_{w}\right]$$

where

(3) $$a_{i}=\left[b_{1},b_{2},\cdots ,b_{j},\cdots ,b_{l}\right]$$

$a_{i}$ represents the trimmed bytes in packet $p_{i}$ and $b_{j}$ represents j-th byte in $a_{i}$.

#### 3.3.2. Normalization

Prior to being fed into the model, it is essential to normalize all data. One-hot encoding is utilized in some studies [15,16]. One byte is encoded into a 256-dimensional vector, and its effectiveness is empirically validated. Nevertheless, the increase in data dimensionality also incurs additional computational expenses. Some works [16,33] just treat a byte as a numerical value within the range of [0, 255] and normalize it by dividing it by 255. The packet header bytes contain important flags, lengths, or other information and are not always expressed in exactly one byte. For example, in the IPv4 protocol, two bytes are used for total length, and in the TCP protocol, two bytes are used for port numbers. Thus, simply considering one byte as a number within the range of [0, 255] poses challenges in learning the representative packet features.

Here, we employ a more fine-grained approach to normalization by integrating an embedding layer to encode the header bytes into a higher-dimensional space and dividing the other bytes by 255. Then, we concatenate the two parts. The embedding dimension can be selected from 1 to 256, and we choose 16 as a compromise. For a TCP packet, the header bytes typically include 14 bytes for the Ethernet protocol, 20 bytes for the IPv4 protocol, and 20 bytes for the TCP protocol. Therefore, the dimension of the embedding vector for the head bytes is 544 ((54−20)×16). We concatenate all normalized values and reshape them into a 45×45 matrix, padding with some zeros.

Finally, the dimension of $a_{i}$ is adjusted to 45×45, i.e., $a_{i}\in R^{45\times 45}$.

#### 3.3.3. Model Structures

**Figure 4:**
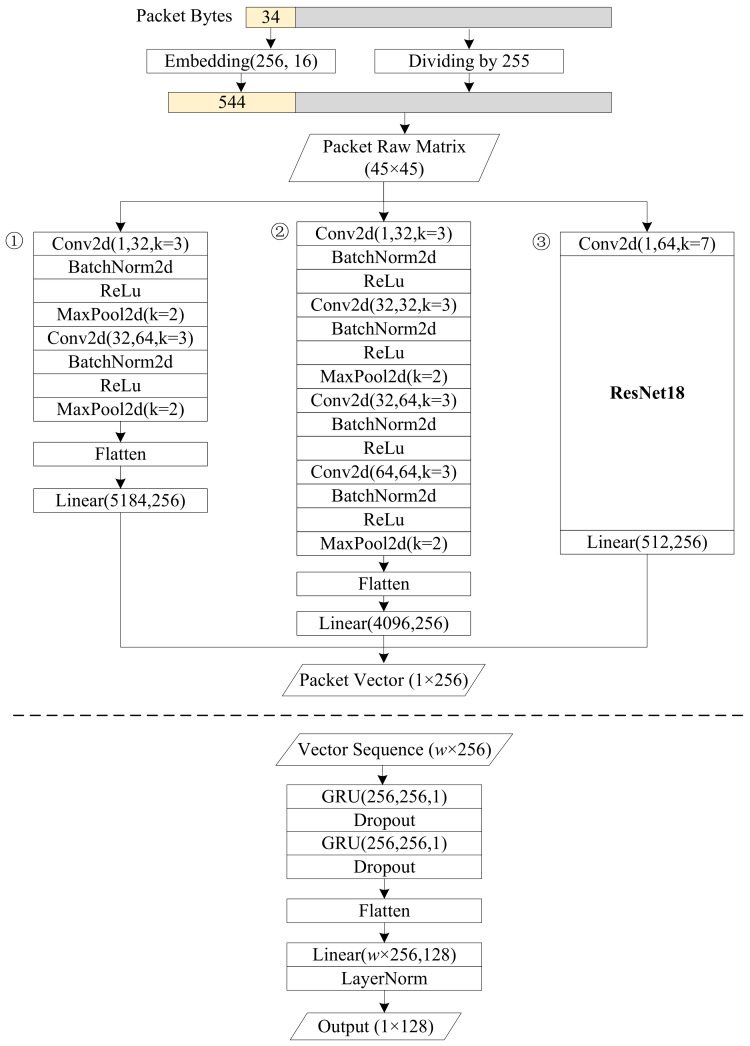
The hierarchical model structure of CHCG.

We use 2D-CNN and GRU-based RNN to learn the spatiotemporal representation of $P_{l}^{\left(w\right)}$. The hierarchical model structure is shown in Figure 4. In this paper, we utilize three CNN models: a basic model with only two convolutional layers, a relatively deeper model with four convolutional layers, and ResNet18, a classic model in the field of computer vision. CNN converts the raw data of a single packet into a vector, and a vector sequence can be formed after all packets in the window have been processed. The sequence is input into the two-layer GRU networks. The output of the entire model is a 128-dimensional vector representing the spatiotemporal features of the packets in the detection window.

#### 3.3.4. Packet Spatial Feature Extraction

CNN has demonstrated outstanding performance in the field of computer vision and has subsequently been expanded to various domains. In comparison to deep neural networks (DNN), CNN decreases the quantity of weights and computational complexity, thereby enhancing the efficiency of training and prediction. We utilize CNN for the extraction of packet spatial features. To evaluate the impact of the depth of CNN, three CNN models are employed. The first model consists of two standard convolutional blocks, which include convolution, batch normalization, rectified linear unit (ReLU), and max-pooling operations. The second model exhibits increased depth by incorporating four convolutional blocks and eliminating two maxpool layers. The third model utilized in this study is the classical ResNet18 model, which has been adapted to meet the specific requirements by modifying its input and fully connected layers. Subsequently, the vector generated by the CNN will be flattened and its dimensionality reduced to 256 through a linear layer.

#### 3.3.5. Temporal Feature Extraction

RNN plays an important role in learning temporal features. LSTM [37] and GRU [38] are the most commonly used RNN units. Compared to LSTM, GRU has a simpler structure, resulting in lower computational costs. However, GRU can achieve performance as good as LSTM most of the time. Two GRU blocks are used in our model, each consisting of a 256-unit GRU layer and a dropout layer.

After learning the temporal feature, we add a flatten layer to normalize and reshape the feature. Afterward, we utilize another linear layer to decrease the dimension to 128. Finally, we obtain the spatiotemporal feature vector $v\in R^{128}$ of the windowed packet sequence.

### 3.4. Training and Detecting

#### 3.4.1. Contrastive Loss

Our goal is to train a representation model so that the representation vectors of the same class traffic fall into a compact cluster, while the representation vectors of different class traffic are far from each other. We assume that the training dataset has been split into minibatches with the size of *N*, and each instance in the batch has been projected to a feature vector $v_{i}\in R^{128}$. The corresponding label is $y_{i}\in 0,1$, where 0 indicates a benign traffic instance and 1 indicates an attack instance. The supervised contrastive loss function used in our method is shown as Equation (4).

(4) $$\begin{array}{c} L_{ij}=-\log\frac{1\left(y_{i}=y_{j}\right)\exp\left(v_{i}^{T}v_{j}/\tau \right)}{\exp\left(v_{i}^{T}v_{j}/\tau \right)+\sum_{k=1}^{N}1\left(y_{i}\neq y_{k}\right)\exp\left(v_{i}^{T}v_{k}/\tau \right)}\\ L=\frac{1}{N(N-1)}\sum_{i=1}^{N}\sum_{j\neq i}^{N}L_{ij} \end{array}$$

Here, $\tau \in R^{+}$ represents a scalar temperature parameter, and $L_{ij}$ is the loss of the positive pair $\left(v_{i},v_{j}\right)$. 1(.) is the indicator function, and it equals 1 if the condition is satisfied. A positive pair is defined as a vector pair with the same label. Then, we sum up all positive pairs to calculate the batch loss L. By minimizing the loss function, we aim to maximize the similarity among representations of the same class and minimize the similarity among representations from different classes.

#### 3.4.2. Detecting

The idea is to calculate the similarity between the representation of the test instance $v^{\mathrm{test}\;}$ and the average vector $\bar{v}$ of benign representations to determine whether the test instance is benign or not.

First, we generate all the benign representations. Then, we use density-based spatial clustering of applications with noise (DBSCAN) algorithm to remove outliers. Although most benign traffic representations are in a small cluster, some instances are still close to the boundary of intrusion traffic. It is reasonable to remove outliers for higher precision. DBSCAN is widely used in many real-world applications due to its simplicity, efficiency, and robustness [39]. For the rest of the vectors, we calculate the average vector $\bar{v}$ by Equation (5).

(5) $$\bar{v}=\frac{1}{n}\sum_{i}\left(\frac{v_{i}}{{∥v_{i}∥}_{2}}\right)$$

where ${∥\cdot ∥}_{2}$ denotes the L2-norm function. The cosine similarity estimator is employed to quantify the similarity between the test vector and the average benign vector.

(6) $$\mathrm{sim}=\frac{{\bar{v}}^{T}v^{\mathrm{test}\;}}{∥\bar{v}{∥}_{2}\times {∥v^{\mathrm{test}\;}∥}_{2}}$$

The similarity sim ranges from 0 to 1, and a value close to 1 indicates high similarity. Finally, we can use a threshold *t* to determine the category of the test instance. *t* needs to be manually selected according to specific requirements. A higher threshold leads to more precise detection results, but it also allows more malicious traffic to bypass detection. Conversely, a lower threshold increases the recall rate but also results in a higher false positive rate (FPR).

## 4. Experiments

### 4.1. Dataset and Experimental Setup

We implemented CHCG on the PyTorch platform [40]. We ran all the experiments on a computer with an Intel(R) Xeon(R) Gold 6248R CPU, a NVIDIA GeForce RTX 3090 GPU and Windows 10 OS. The datasets used in the experiments include CIC-IDS2017 [19] and CSE-CIC-IDS2018 [20], which are relatively lately published and have been frequently used by recent works.

The CIC-IDS2017 dataset was created by the Canadian Institute of Cybersecurity in 2017 and introduced by Sharafaldin et al. [19]. They tried to generate realistic background traffic and used B-Profile system to profile the abstract behavior of human interactions. The naturalistic benign traffic provides a better assessment of the performance in the real world. They implemented several common attacks including BruteForce FTP, BruteForce SSH, DoS, Heartbleed, WebAttack, Infiltration, Botnet, and DDoS. CSE-CIC-IDS2018 was created with a similar method but in a more complicated network topology. The two datasets both provide raw PCAP data, up to 48.8 GB and 444.5 GB, respectively.

Liu et al. analyzed the errors [41] present in both datasets. They analyzed the inconsistency of the dataset labels with the actual attack behavior and provided a modified dataset. However, what they provide is still feature-ready data that cannot be directly used in the experiments. In fact, we split and labeled the PCAP data based on the attack paths, attack times, and other information from official documents, rather than the CSV files provided. According to our analysis, we found some issues that are consistent with the findings of Liu (2022) [41]. For example, in CSE-CIC-IDS2018, the execution description of BruteForce FTP can not be matched with the PCAP data, so we removed it from the attack list. Infiltration was divided into two steps: first, the attacker compromises an internal host, and then uses that host to launch a network scanning attack. We extracted the traffic from the latter part as Infiltration. Liu et al. also raises the issue of empty payloads, which we did not distinguish and categorized them all as attacks.

Overall, the dataset we generated by extracting from the original PCAP file eliminates most of the problems in the feature-ready CSV dataset. While both datasets still suffer from the problem that some of the traffic does not exactly match the real attack process, it is reasonable to use them to evaluate our method.

The details of the CIC-IDS2017 and CSE-CIC-IDS2018 datasets at the IP pair granularity are summarized in Table 2 and Table 3, including the numbers of total, train, and test samples. Obviously, there is a significant imbalance in both CIC-IDS2017 and CSE-CIC-IDS2018, which is consistent with the real world. The proportion of malicious traffic is much smaller than that of benign traffic, and the proportion of different types of malicious traffic also varies significantly.

It is reasonable to assume that we collected a mass of benign traffic and several categories of malicious traffic. The collected traffic was then labeled to train the model. In the experiments, we divided the attacks into known and unknown attacks based on the volume of their sample numbers. For CIC-IDS2017, we split the benign samples and known attack samples into the training and testing sets with a 1:1 ratio. The known attacks include BruteForce-FTP, BruteForce-SSH, PortScan, DDoS LOIC, DoS Hulk, and DoS GoldenEye. Other attacks were considered unknown and were only used during the testing phase.

For CSE-CIC-IDS2018, the number of samples for benign and certain attacks is excessive, and it was unnecessary to use all the samples. The ratio of division between the training set and testing set is also 1:1. However, in the training and test sets, only 1,000,000 benign samples were randomly selected, and the maximum number of samples per attack was limited to 100,000. The known attacks include DDoS-HOIC, DDoS LOIC-HTTP, Botnet, SSH-BruteForce, DoS-Hulk, DDoS-LOIC-UDP, and Infiltration.

To evaluate the performance of the proposed CHCG, we used accuracy (AC), precision (PR), recall (RC), and F1-score (F1) as the metrics. Concerning intrusion detection tasks, all results correspond to the following four outcomes: (1) TP (true positive): malicious samples correctly identified as malicious; (2) TN (true negative): benign samples correctly identified as benign; (3) FP (false positive): benign samples incorrectly identified as malicious; (4) FN (false negative): malicious samples incorrectly identified as benign. Therefore, the formulas for the above metrics are defined as follows.

(7) $$\;\mathrm{precision}\;=\frac{TP}{TP+FP}.$$

(8) $$\;\mathrm{recall}\;=\frac{TP}{TP+FN}.$$

(9) $$\mathrm{accuracy}=\frac{TP+TN}{TP+TN+FP+FN}.$$

(10) $$F1\text{-}\mathrm{score}=\frac{2\times \;\mathrm{Precision}\;\times \;\mathrm{Recall}\;}{\;\mathrm{Precision}\;+\;\mathrm{Recall}\;}.$$

These metrics are heavily influenced by data imbalances. Due to the high proportion of benign traffic, even with high accuracy, a significant number of benign samples may be incorrectly classified as malicious, which results in low precision. For attacks involving a small number of samples, they have little impact on the overall metrics. Therefore, macro-averages and weighted-averages of the above metrics were used to evaluate the overall detection performance. Unknown-averages were used to evaluate the detection effectiveness of unknown attacks.

Taking precision as an example, assuming there are $n_{m}$ types of attacks and $n_{u}$ types of unknown attacks, and each type of attack has $M_{i}$ samples, macro-average precision, weighted-average precision, and unknown-average precision are calculated as follows:

(11) $$\;\mathrm{Macro}\text{\_}\mathrm{PR}=\frac{1}{n_{m}}\sum_{i=1}^{n_{m}}{\mathrm{PR}}_{i}$$

(12) $$\;\mathrm{Weighted}\text{\_}\mathrm{PR}=\frac{1}{\sum_{i=1}^{n_{m}}M_{i}}\sum_{i=1}^{n_{m}}\left({\mathrm{PR}}_{i}\times M_{i}\right)$$

(13) $$\;\mathrm{Unknown}\text{\_}\mathrm{PR}=\frac{1}{\sum_{i=1}^{n_{u}}M_{i}}\sum_{i=1}^{n_{u}}\left({\mathrm{PR}}_{i}\times M_{i}\right)$$

Each attack contributes equally to macro-averages and is not affected by the sample size, so classes with low scores have a greater impact. Weighted-averages increase the impact of sample size, while unknown-averages are similar but only for unknown attacks.

### 4.2. Detection Results on Different Datasets

We trained and tested the three models (different in the CNN module) separately on CIC-IDS2017 and CSE-CIC-IDS2018. We segmented the PCAP data at the IP pair granularity, and each sequence has 20 packets. In addition, detecting unknown attacks is a key challenge. We evaluated the method’s ability to detect unknown attacks by evaluating its effectiveness in detecting attack classes that were not part of the training set.

The detection results for the CIC-IDS2017 dataset are presented in Table 4. We present the recall, accuracy, and F1-score. Recall reflects CHCG’s detection ability for each attack category, accuracy measures the detection effect on all samples, and the F1-score combines precision and recall. The proposed method demonstrates an improved overall detection effect on CIC-IDS2017, achieving recall and accuracy rates above 99% for most categories. Specifically, CHCG exhibits high F1-scores for unknown attacks such as WebAttack-BruteForce, WebAttack-XSS, and WebAttack-SQL Injection, but it performs poorly in detecting Infiltration and Botnet. We find that the infiltration contains only 39 flows, each with a long duration and a high volume of packets. This increases the difficulty of distinguishing benign streams after the detection window is split. The utilization of ResNet18 drastically improved the detection of Infiltration and Botnet, achieving recall rates of 88.67% and 91.85%. This indicates that deeper CNN modules can augment the feature extraction ability and improve the accuracy of detection.

The low F1-score can be attributed to the significant proportion of benign samples. Despite achieving an overall detection accuracy of 99.9%, the number of misclassified benign samples remains significant, which results in a low detection precision for specific attacks. The weighted F1-score, which takes into account the effect of the number of samples, is at a higher level (above 97% using ResNet18).

The detection results for the CSE-CIC-IDS2018 dataset are shown in Table 5. Except for SQL injection, CHCG shows a high F1-score for both known and unknown attacks. The weighted accuracy reaches 99.9%, indicating a strong detection capability for both benign and malicious samples. Unknown attacks can also be effectively detected. The models achieve a recall of 100%, which means that all unknown attack samples were detected, with an unknown-F1-score of up to 96.77%.

The enhanced detection of unknown attacks in the experiments can be attributed to two primary factors. Firstly, there exist certain resemblances among DoS subclasses, which allow the attacks in the training set to contribute to acquiring knowledge about the characteristics of unknown attacks. Secondly, the introduction of contrastive loss in this study effectively segregates benign samples from malicious samples in the representation space. This process not only generates a more condensed cluster of benign representations but also ensures that representations of unknown samples are distanced from the core of the cluster, facilitating effective recognition.

### 4.3. Detection Results with Different Hyperparameters

There are two essential hyperparameters in data processing and training, the detection window size *w* and the scaling temperature factor τ.

#### 4.3.1. Detection Results with Different Window Size *W*

The window size plays a crucial role in determining the frequency of detection; a smaller *w* results in faster detection frequency and reduced packet storage pressure on the IDS. However, this may lead to increased computational pressure on the model and difficulty in effectively representing some attacks. Conversely, a larger *w* leads to higher storage pressure and potential introduction of excessive noise, thereby increasing the challenge in representation.

We used a 2-Layer CNN + GRU model to test the performance on different window size of 10, 20, and 30, and CSE-CIC-IDS2018 was chosen as the dataset. Table 6 shows the test results. It can be seen that when *w* is 30, the detection performance of CHCG is the worst, and unknown-average-F1 is about 3% lower than w=10. When the parameter *w* is set to 10 and 20, CHCG can obtain better detection results. Both known and unknown attacks can achieve high recall rates and F1-scores.

**Figure 5:**
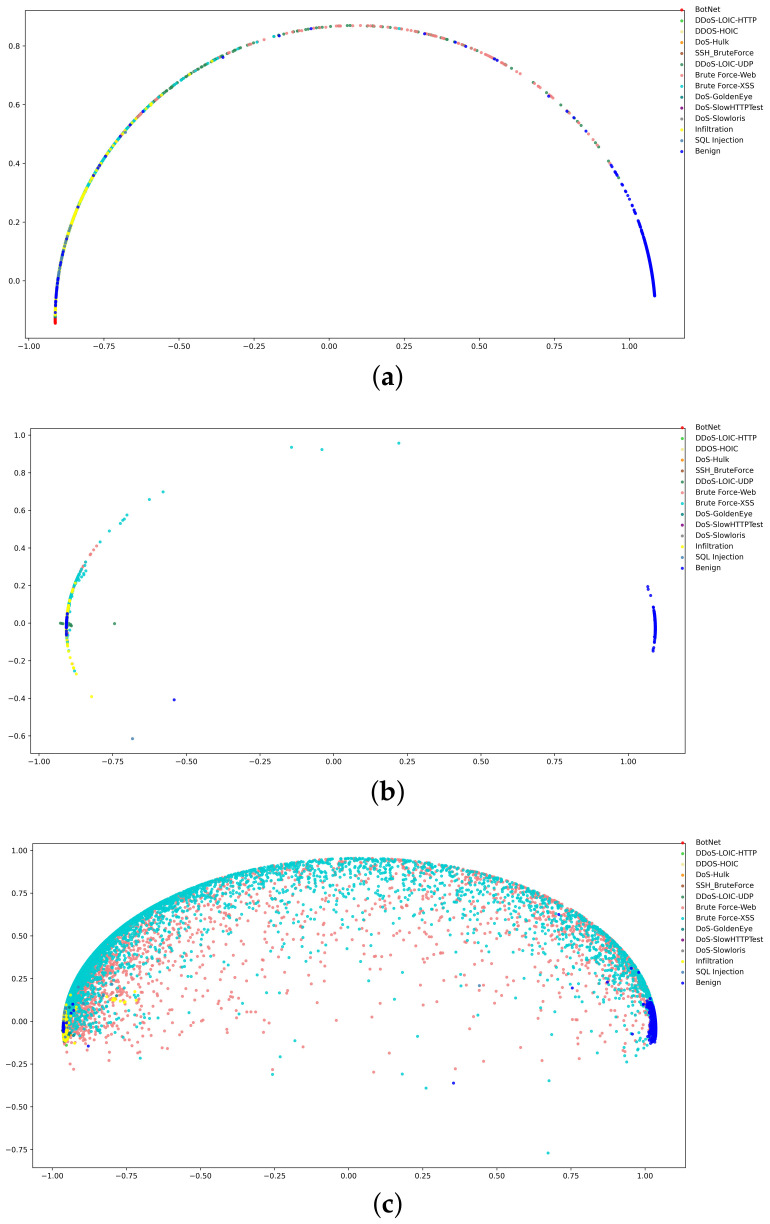
Distribution of traffic representation using PCA visualization. (**a**) w = 10. (**b**) w = 20. (**c**) w = 30.

Figure 5 illustrates the distribution of sample representation vectors visualized after PCA dimensionality reduction. When *w* is set to 30, BruteForce-XSS and BruteForce-Web show dispersion and are partially mixed with benign traffic, so a certain number of malicious samples are classified as benign. When *w* is taken as 10 and 20, there is a significant difference in the distribution of benign and attack traffic, making the detection of malicious traffic easy. Contrastive loss plays a key role in separating benign and malicious samples, and the large margin between the two makes it easy to detect unknown attacks as well.

#### 4.3.2. Detection Results with Different τ

As indicated in preceding sections, the scaling factor serves the purpose of equilibrating the intra-class and inter-class distances [42]. A smaller scaling factor leads to a quicker change in the contrastive loss, an increased emphasis on hard samples, a higher penalty for ambiguous samples near the boundary, and a finer delineation of the class boundary. A larger scaling factor results in a slower rate of change in the contrastive loss, a reduced penalty for ambiguous samples near the boundary, and a smoother class boundary.

We tested the CHCG with temperature factors of 0.03, 0.05, 0.07, 0.1, 0.15, 0.2, and 0.5 on CSE-CIC-IDS2018. As shown in Table 7, the performance of the model remains relatively stable as τ increases, indicating that the proposed model has a strong representation capability. However, in the process of experiments, we observed that the distributions of the representations after dimensional reduction exhibit a clear trend. The clusters are sparsest at τ = 0.03 and become progressively more compact as τ increases, which is consistent with the findings of the work [42]. We recommend setting τ to 0.1 for optimal performance.

### 4.4. Ablation Study

CHCG is structured as a hierarchical architecture combining CNN and GRU with an Embedding layer aimed at improving the encoding of protocol header bytes. This section tests the Embedding layer used and the hierarchical CNN and GRU structures.

#### 4.4.1. Embedding Layer

Experiments were conducted on the CSE-CIC-IDS2018 dataset using consistent parameter configurations after excluding the Embedding layer. The results of the experiments are displayed in the Table 8. After removing the Embedding layer, the detection capability of some attacks is significantly reduced, including DDoS-LOIC-UDP, Brute Force-XSS, and Brute Force-Web. Unknown-average recalls decreased by 1.12%, and Weighted-average F1-score decreased by 1.54%.

The experimental results show that the performance of CHCG is improved by the addition of the Embedding layer.

This shows that it is necessary to enhance the representation of protocol header bytes. Header bytes contain important flags, lengths, or other information, and it is unreasonable to simply treat a byte as a number in the range [0, 255]. In addition, there is no clear semantic connection between the values corresponding to bytes, and similar values do not necessarily mean that they represent similar information. For example, the two bytes representing port numbers, 80 and 81, are far apart semantically, although the difference is very small numerically.

The complexity of network protocols presents challenges in evaluating the Embedding layer. Other network protocols, such as UDP and ICMP, may not always meet the practical requirements for the number of embedding bytes. In addition, there are optional fields in the IP and TCP protocol headers, which contribute to the expansion of the protocol header bytes. The utilization of encryption protocols further increases the number of protocol header bytes, making it challenging to find a unified embedding approach. The evaluation carried out in this paper mainly uses a standard TCP protocol packet as a template. It selects 34 bytes for embedding, excluding the MAC address and IP address. However, there may be a certain degree of non-adaptation for other protocols.

#### 4.4.2. The Hierarchical CNN and GRU Model

Experiments were conducted on the CSE-CIC-IDS2018 dataset using a consistent parameter configuration with the CNN or GRU module removed. We used the encoder of a vanilla autoencoder to replace the CNN module, which consists of two linear layers. The experimental results are displayed in the Table 9. After using linear layer instead of CNN, the recall rate of malicious traffic remains high, but the detection effect of DDoS LOIC in known attacks is reduced to some extent. In addition, the F1-score also decreased to a certain extent, indicating that there were more misscores for benign samples. The group with the GRU removed had a larger drop in recall for unknown attacks, down 2.5%. However, the decrease of F1-score is small, indicating that the detection ability of benign samples is more accurate. The experiment verifies the practicability of the CNN and gated cycle unit (GRU) in enhancing the feature description ability of the model.

## 5. Conclusions

In this paper, we propose CHCG, a contrastive learning-based end-to-end intrusion detection framework with hierarchical CNN and GRU networks to extract features from raw traffic. The network architecture combines hierarchical CNN and GRU to autonomously extract spatiotemporal features from sequences of packets, eliminating the need for manual feature engineering. The inclusion of an embedding layer enhances the encoding of protocol header bytes, as empirically demonstrated to significantly improve detection precision and F1 scores. Contrastive learning improves the model’s capacity to encode benign network traffic into a compact cluster while also amplifying the differentiation between benign and malicious representations. This, in turn, aids in the expansion of the margins of the decision boundary. The proposed framework is assessed utilizing commonly employed datasets, namely, CIC-IDS2017 and CSE-CIC-IDS2018. The experiments demonstrate that our method can attain a detection accuracy of 99.9% for known attacks, thus achieving state-of-the-art performance. Our method also achieved a recall rate of over 95% for all unknown attack samples. Our future work will focus on achieving a fine-grained classification of malicious network traffic while also maintaining the capability to identify unknown attacks.

## Figures and Tables

**Table 1:** Summary of the related works.

	Methods		End-to-End	Unknown Attack Detection
Signature-based	Preconfigured Rules or Signatures [6,7]		No	No
Anomaly-based	Traditional machine learning	SVM [9], DT [10], KNN [11]	No	No
	Deep Learning	HELAD [13,23]	No	No
		[12], Kitsune [21,31,32]	No	Yes
		HAST-IDS [15], PBCNN [16], FlowPic [24,33,34]	Yes	No
		**The proposed CHCG**	Yes	Yes

**Table 2:** Details of windowed sequences in CIC-IDS2017 for w=20.

	Traffic Category	Total	Train	Test
**Known**	Benign	1,073,124	536,562	536,562
	DDoS LOIC	128,060	64,030	64,030
	DoS Hulk	124,695	62,348	62,348
	PortScan	32,406	16,203	16,203
	BruteForce-SSH	14,025	7013	7013
	BruteForce-FTP	11,159	5580	5580
	DoS GoldenEye	10,561	5281	5281
**Unknown**	Infiltration	7445	0	7445
	DoS Slowloris	4757	0	4757
	DoS Slowhttptest	3966	0	3966
	WebAttack-BruteForce	3026	0	3026
	Botnet	1304	0	1304
	WebAttack-XSS	963	0	963
	WebAttack-SQL Injection	31	0	31
	All	**1,415,522**	**697,015**	**718,507**

**Table 3:** Details of windowed sequences in CSE-CIC-IDS2018 for w=20.

	Traffic Category	w = 10			w = 20			w = 30		
		Train	Test	Total	Train	Test	Total	Train	Test	Total
**Known**	Benign	205,769,000	1,000,000	1,000,000	92,834,300	1,000,000	1,000,000	68,091,800	1,000,000	1,000,000
	DDoS-LOIC-UDP	9,852,201	100,000	100,000	4,926,098	100,000	100,000	3,284,063	100,000	100,000
	DoS-Hulk	4,069,009	100,000	100,000	2,034,504	100,000	100,000	1,356,335	100,000	100,000
	SSH-BruteForce	862,013	100,000	100,000	431,006	100,000	100,000	287,337	100,000	100,000
	Botnet	734,252	100,000	100,000	367,126	100,000	100,000	244,746	100,000	100,000
	DDoS LOIC-HTTP	664,234	100,000	100,000	332,118	100,000	100,000	221,402	100,000	100,000
	DDOS-HOIC	241,474	100,000	100,000	120,736	60,368	60,368	80,485	40,243	40,242
	Infiltration	79,443	39,722	39,721	39,756	19,878	19,878	26,363	13,182	13,181
**Unknown**	DoS-GoldenEye	51,828	0	51,828	25,917	0	25,917	17,272	0	17,272
	DoS-SlowHTTPTest	42,219	0	42,219	21,109	0	21,109	14,073	0	14,073
	DoS-Slowloris	21,386	0	21,386	10,693	0	10,693	7128	0	7128
	Brute Force-XSS	13,648	0	13,648	6823	0	6823	4549	0	4549
	Brute Force-Web	12,433	0	12,433	6216	0	6216	4143	0	4143
	SQL Injection	65	0	65	33	0	33	19	0	19
	All	**222,413,205**	**1,639,722**	**1,781,300**	**101,156,435**	**1,580,246**	**1,651,037**	**73,639,715**	**1,553,424**	**1,600,607**

**Table 4:** Results using CIC-IDS2017.

	Category	2-Layer CNN			4-Layer CNN			ResNet18		
		RC	AC	F1	RC	AC	F1	RC	AC	F1
**Known**	DDoS LOIC	1.0000	0.9991	0.9958	1.0000	0.9988	0.9945	1.0000	0.9985	0.9932
	DoS Hulk	1.0000	0.9991	0.9957	1.0000	0.9988	0.9944	1.0000	0.9985	0.9930
	PortScan	1.0000	0.9990	0.9836	1.0000	0.9987	0.9786	1.0000	0.9984	0.9737
	BruteForce-SSH	0.9999	0.9990	0.9629	1.0000	0.9987	0.9520	1.0000	0.9983	0.9413
	BruteForce-FTP	1.0000	0.9990	0.9538	1.0000	0.9987	0.9404	1.0000	0.9983	0.9273
	DoS GoldenEye	1.0000	0.9990	0.9514	1.0000	0.9987	0.9373	1.0000	0.9983	0.9235
**Unknown**	Infiltration	0.0930	0.9862	0.1596	0.2836	0.9885	0.4115	0.8867	0.9967	0.8848
	DoS Slowloris	1.0000	0.9990	0.9463	1.0000	0.9986	0.9308	1.0000	0.9983	0.9158
	DoS Slowhttptest	1.0000	0.9990	0.9362	1.0000	0.9986	0.9181	1.0000	0.9983	0.9006
	WebAttack-BruteForce	1.0000	0.9990	0.9181	1.0000	0.9986	0.8954	1.0000	0.9983	0.8736
	Botnet	0.2864	0.9976	0.3119	0.8675	0.9984	0.6703	0.9185	0.9982	0.6536
	WebAttack-XSS	1.0000	0.9990	0.7808	1.0000	0.9986	0.7313	1.0000	0.9983	0.6874
	WebAttack-SQL Injection	1.0000	0.9990	0.0391	1.0000	0.9986	0.0302	1.0000	0.9983	0.0245
	**Macro-average**	0.8753	0.9979	0.7642	0.9347	0.9979	0.7988	0.9850	0.9982	0.8225
	**Weighted-average**	0.9590	0.9985	0.9478	0.9699	0.9983	0.9560	0.9949	0.9984	0.9714
	**Unknown-average**	0.6477	0.9944	0.6261	0.7418	0.9951	0.7190	0.9563	0.9978	0.8730

**Table 5:** Results using CSE-CIC-IDS2018.

	Category	2-Layer CNN			4-Layer CNN			ResNet18		
		RC	AC	F1	RC	AC	F1	RC	AC	F1
**Known**	DDoS-LOIC-UDP	1.0000	0.9990	0.9951	1.0000	0.9990	0.9951	1.0000	0.9990	0.9953
	DoS-Hulk	1.0000	0.9990	0.9951	1.0000	0.9990	0.9951	1.0000	0.9990	0.9953
	SSH-BruteForce	1.0000	0.9990	0.9951	1.0000	0.9990	0.9951	1.0000	0.9990	0.9953
	Botnet	1.0000	0.9990	0.9951	1.0000	0.9990	0.9951	1.0000	0.9990	0.9953
	DDoS LOIC-HTTP	1.0000	0.9990	0.9951	1.0000	0.9990	0.9951	1.0000	0.9990	0.9953
	DDOS-HOIC	1.0000	0.9989	0.9919	1.0000	0.9989	0.9919	1.0000	0.9990	0.9922
	Infiltration	1.0000	0.9989	0.9758	1.0000	0.9989	0.9756	1.0000	0.9989	0.9767
**Unknown**	DoS-GoldenEye	1.0000	0.9989	0.9814	1.0000	0.9989	0.9812	1.0000	0.9989	0.9821
	DoS-SlowHTTPTest	1.0000	0.9989	0.9773	1.0000	0.9989	0.9770	1.0000	0.9989	0.9781
	DoS-Slowloris	1.0000	0.9989	0.9561	1.0000	0.9989	0.9557	1.0000	0.9989	0.9577
	Brute Force-XSS	1.0000	0.9989	0.9329	1.0000	0.9989	0.9322	1.0000	0.9989	0.9352
	Brute Force-Web	1.0000	0.9989	0.9268	1.0000	0.9989	0.9261	1.0000	0.9989	0.9294
	SQL Injection	1.0000	0.9989	0.0594	1.0000	0.9989	0.0588	1.0000	0.9989	0.0616
	**Macro-average**	1.0000	0.9989	0.9059	1.0000	0.9989	0.9057	1.0000	0.9990	0.9069
	**Weighted-average**	1.0000	0.9990	0.9911	1.0000	0.9990	0.9910	1.0000	0.9990	0.9914
	**Unknown-average**	1.0000	0.9989	0.9665	1.0000	0.9989	0.9661	1.0000	0.9989	0.9677

**Table 6:** Detection results with different window size on CSE-CIC-IDS2018.

	Traffic Category	w = 10			w = 20			w = 30		
		RC	AC	F1	RC	AC	F1	RC	AC	F1
**Known**	DDoS-LOIC-UDP	1.0000	0.9990	0.9947	1.0000	0.9990	0.9951	1.0000	0.9990	0.9956
	DoS-Hulk	1.0000	0.9990	0.9947	1.0000	0.9990	0.9951	1.0000	0.9990	0.9956
	SSH-BruteForce	1.0000	0.9990	0.9947	1.0000	0.9990	0.9951	1.0000	0.9990	0.9956
	Botnet	1.0000	0.9990	0.9947	1.0000	0.9990	0.9951	1.0000	0.9990	0.9956
	DDoS LOIC-HTTP	1.0000	0.9990	0.9947	1.0000	0.9990	0.9951	1.0000	0.9990	0.9956
	DDOS-HOIC	1.0000	0.9990	0.9947	1.0000	0.9989	0.9919	1.0000	0.9990	0.9891
	Infiltration	0.9999	0.9990	0.9868	1.0000	0.9989	0.9758	1.0000	0.9989	0.9673
**Unknown**	DoS-GoldenEye	1.0000	0.9990	0.9899	1.0000	0.9989	0.9814	1.0000	0.9989	0.9749
	DoS-SlowHTTPTest	1.0000	0.9990	0.9876	1.0000	0.9989	0.9773	1.0000	0.9989	0.9693
	DoS-Slowloris	1.0000	0.9990	0.9758	1.0000	0.9989	0.9561	1.0000	0.9989	0.9412
	Brute Force-XSS	1.0000	0.9990	0.9627	1.0000	0.9989	0.9329	0.9560	0.9987	0.8885
	Brute Force-Web	1.0000	0.9990	0.9592	1.0000	0.9989	0.9268	0.9549	0.9987	0.8801
	SQL Injection	1.0000	0.9989	0.1093	1.0000	0.9989	0.0594	1.0000	0.9989	0.0409
	**Macro-average**	1.0000	0.9990	0.9184	1.0000	0.9989	0.9059	0.9931	0.9989	0.8945
	**Weighted-average**	1.0000	0.9990	0.9919	1.0000	0.9990	0.9911	0.9994	0.9990	0.9910
	**Unknown-average**	1.0000	0.9990	0.9814	1.0000	0.9989	0.9665	0.9918	0.9989	0.9511

**Table 7:** Performance on CSE-CIC-IDS2018 with different τ.

τ	Macro RC	Macro F1	Weighted F1	Unknown F1
0.03	1.0000	0.8990	0.9886	0.9573
0.05	0.9984	0.9034	0.9890	0.9643
0.07	1.0000	0.9018	0.9896	0.9611
0.1	1.0000	0.9059	0.9911	0.9665
0.15	0.9456	0.8714	0.9872	0.9268
0.2	1.0000	0.9032	0.9902	0.9630
0.5	1.0000	0.9018	0.9897	0.9612

**Table 8:** Performance on CSE-CIC-IDS2018 with and without the Embedding layer.

	Category	with Embedding			without Embedding		
		RC	AC	F1	RC	AC	F1
**Known**	DDoS-LOIC-UDP	1.0000	0.9990	0.9951	0.9073	0.9886	0.9424
	DoS-Hulk	1.0000	0.9990	0.9951	1.0000	0.9981	0.9909
	SSH-BruteForce	1.0000	0.9990	0.9951	1.0000	0.9981	0.9909
	Botnet	1.0000	0.9990	0.9951	1.0000	0.9981	0.9909
	DDoS LOIC-HTTP	1.0000	0.9990	0.9951	1.0000	0.9981	0.9909
	DDOS-HOIC	1.0000	0.9989	0.9919	1.0000	0.9980	0.9851
	Infiltration	1.0000	0.9989	0.9758	0.9999	0.9979	0.9559
**Unknown**	DoS-GoldenEye	1.0000	0.9989	0.9814	0.9999	0.9980	0.9659
	DoS-SlowHTTPTest	1.0000	0.9989	0.9773	1.0000	0.9980	0.9585
	DoS-Slowloris	1.0000	0.9989	0.9561	1.0000	0.9979	0.9213
	Brute Force-XSS	1.0000	0.9989	0.9329	0.9052	0.9972	0.8331
	Brute Force-Web	1.0000	0.9989	0.9268	0.9765	0.9977	0.8601
	SQL Injection	1.0000	0.9989	0.0594	1.0000	0.9979	0.0328
	**Macro-average**	1.0000	0.9989	0.9059	0.9838	0.9972	0.8784
	**Weighted-average**	1.0000	0.9990	0.9911	0.9845	0.9966	0.9757
	**Unknown-average**	1.0000	0.9989	0.9665	0.9888	0.9979	0.9344

**Table 9:** Results of CNN and GRU ablation experiments.

	Category	Linear layers + GRU		CNN without GRU		CNN + GRU	
		RC	F1	RC	F1	RC	F1
**Known**	DDoS LOIC	0.9844	0.9893	1.0000	0.9954	1.0000	0.9951
	DoS Hulk	1.0000	0.9897	1.0000	0.9954	1.0000	0.9951
	PortScan	1.0000	0.9897	1.0000	0.9954	1.0000	0.9951
	BruteForce-SSH	1.0000	0.9897	1.0000	0.9954	1.0000	0.9951
	BruteForce-FTP	1.0000	0.9897	1.0000	0.9954	1.0000	0.9951
	DoS GoldenEye	1.0000	0.9831	1.0000	0.9923	1.0000	0.9919
**Unknown**	Infiltration	1.0000	0.9503	1.0000	0.9770	1.0000	0.9758
	DoS Slowloris	1.0000	0.9616	1.0000	0.9823	1.0000	0.9814
	DoS Slowhttptest	1.0000	0.9532	1.0000	0.9784	1.0000	0.9773
	WebAttack-BruteForce	1.0000	0.9117	1.0000	0.9582	1.0000	0.9561
	Botnet	1.0000	0.8682	0.8344	0.8466	1.0000	0.9329
	WebAttack-XSS	1.0000	0.8571	0.8970	0.8764	1.0000	0.9268
	WebAttack-SQL Injection	1.0000	0.0291	0.9677	0.0604	1.0000	0.0594
	**Macro-average**	0.9988	0.8817	0.9769	0.8960	1.0000	0.9059
	**Weighted-average**	0.9976	0.9817	0.9973	0.9901	1.0000	0.9911
	**Unknown-average**	1.0000	0.9330	0.9750	0.9547	1.0000	0.9665

## Data Availability

These data were derived from the following resources available in the public domain: [CIC-IDS2017: https://www.unb.ca/cic/datasets/ids-2017.html, accessed on 21 March 2024, CSE-CIC-IDS2018: https://www.unb.ca/cic/datasets/ids-2018.html], accessed on 21 March 2024.

## Abbreviations

The following abbreviations are used in this manuscript:

CNN	Convolutional Neural Network
GRU	Gated Recurrent Unit
IoT	Internet of Things
NIDS	Network intrusion detection system
RNN	Recurrent Neural Network
PCA	Principal Component Analysis
TCP	Transmission Control Protocol
IP	Internet Protocol
